# Children’s perspectives on life and well-being after parental intimate partner homicide

**DOI:** 10.1080/20008198.2018.1463796

**Published:** 2018-05-22

**Authors:** Eva Alisic, Arend Groot, Hanneke Snetselaar, Tielke Stroeken, Lieve Hehenkamp, Elise van de Putte

**Affiliations:** a Monash University Accident Research Centre, Monash University, Melbourne, Australia; b Psychotrauma Centre Wilhelmina Children’s Hospital, University Medical Centre Utrecht, Utrecht, The Netherlands; c Wilhelmina Children’s Hospital, University Medical Centre Utrecht, Utrecht, The Netherlands

**Keywords:** Bereavement, child protection, domestic homicide, femicide, foster care, intimate partner violence, qualitative research, traumatic grief, uxoricide, 丧亲, 儿童保护, 家庭凶杀, 杀害妇女, 寄养, 亲密伴侣暴力, 定性研究, 创伤性悲伤, 杀妻者, duelo, protección infantil, homicidio doméstico, femicidio, orfanato, violencia en la pareja, investigación cualitativa, dolor traumático, uxoricida, • Children are willing and able to express their views on key issues related to their life after a parental intimate partner homicide, such as contact with the perpetrating parent and living arrangements.• Children’s views on these issues vary considerably, even between siblings, and should therefore be solicited and taken into account with care.• While most participants were content with their living arrangements, they also talked about distress, conflicts between family members, and lack of continuity in professional support.

## Abstract

**Background:** While there is no doubt that parental intimate partner homicide is associated with strong grief and post-traumatic stress reactions among the children who have been bereaved, there is little in-depth insight into how children and young people see and describe their circumstances and needs.

**Objective:** Our aim was to shed light on children’s and young people’s perspectives on their life after parental intimate partner homicide. In particular, we were interested in how they experienced their living arrangements, social environment, and general well-being.

**Method:** We conducted semi-structured interviews with 23 children and young people (8–24 years old; 15 females and eight males) who had been younger than 18 years when one of their parents killed the other (21 children lost their mother, two children lost their father). We used thematic analysis to synthesize the findings.

**Results:** While most participants were fairly content with themselves and their living arrangements, they also expressed substantial and persistent difficulties, including distress, conflicts between family members, and feelings of unsafety. Most importantly, children’s self-image, their perspectives on their biological parents, and their views on their broader (family) environment varied considerably from participant to participant, and also between siblings.

**Conclusions:** It is unlikely that straightforward guidelines can be given with regard to where the children should live after parental homicide, or whether they should be in contact with the perpetrating parent. Rather, this study’s findings underline the need to explore children’s individual viewpoints carefully during decision-making processes.

## Abstract (es)

**Planteamiento:** Si bien no hay duda de que el homicidio en la pareja se asocia con aflicción y reacciones de estrés postraumático intensas entre los niños que han sufrido un duelo, hay poca información detallada sobre cómo los niños y jóvenes ven y describen sus circunstancias y necesidades.

**Objetivo:** Nuestro objetivo fue arrojar luz sobre las perspectivas de los niños y los jóvenes sobre su vida después del homicidio de uno de los padres a manos del otro En particular, estábamos interesados en cómo han experimentado su situación en cuanto a vivienda, entorno social y bienestar general. Método: Realizamos entrevistas semiestructuradas con 23 niños y jóvenes (de 8 a 24 años, 15 mujeres y 8 hombres) que tenían menos de 18 años cuando uno de sus padres mató al otro (21 niños perdieron a su madre, 2 niños perdieron a su padre). Usamos análisis temáticos para sintetizar los hallazgos.

**Resultados:** Si bien la mayoría de los participantes se sentían bastante satisfechos de sí mismos y de sus condicionesde vida, también expresaron dificultades sustanciales y persistentes, como angustia, conflictos entre los miembros de la familia y sentimientos de inseguridad. Lo que es más importante, la autoimagen de los niños, sus perspectivas sobre sus padres biológicos y sus puntos de vista sobre su entorno más amplio (familiar) variaban considerablemente de un participante a otro, también entre hermanos.

**Conclusiones:** Es poco probable que se puedan dar pautas directas con respecto a dónde deberían vivir los niños después del homicidio de uno de los padres o si deberían estar en contacto con el progenitor perpetrador. Por el contrario, los hallazgos de este estudio subrayan la necesidad de explorar cuidadosamente los puntos de vista de los niños durante los procesos de toma de decisiones.

## Abstract (zh)

背景：毫无疑问，父母遭受亲密伴侣凶杀与丧亲儿童的强烈悲痛和创伤后应激反应相关，但对儿童和年轻人如何看待和描述他们的情况和需求没有深入的了解。

目标：我们的目标是揭示父母遭受亲密伴侣凶杀后的儿童和青少年对其生活的看法。 我们特别感兴趣的是，他们如何体验他们的生活安排，社会环境和一般福利。

方法：我们对23名儿童和青少年（8-24岁，15女，8男）进行了半结构式访谈，他们在不到18岁时父母亲中一位杀害了另一位（21位失去了母亲，2位失去了父亲）。 我们使用主题分析来综合调查结果。

结果：虽然大多数被试对自己及其生活安排相当满意，但他们也表达了大量和持续存在的困难，包括痛苦、家庭成员之间的冲突以及不安全感。 最重要的是，儿童的自我形象，他们对生父母的看法以及他们对更广泛（家庭）环境的看法，在不同被试间，甚至兄弟姐妹之间差异很大。

结论：直接提出指导原则，建议父母遭受凶杀后的孩子应该在何处生活，或者他们是否应该与犯罪父母进行接触，是不太可能的。 相反，这项研究的结果强调了在做决定过程中仔细探索儿童个人观点的必要性。

## 1. Introduction

When one parent kills the other, children are confronted with multiple losses. Not only has one parent died; the other parent is incarcerated, has fled, or has killed themselves (Steeves & Parker, 2007). The children often lose their home, and sometimes also their school and friends. At once, they are the child of a murderer and a victim, and in many cases, they have been directly exposed to the killing or to the crime scene (Alisic, Groot, Snetselaar, Stroeken, & Van de Putte, 2017). In brief, they face a unique combination of trauma, loss and hardship.

Unsurprisingly, concerns have been raised regarding children’s mental health and well-being post-homicide. In particular, strong grief reactions, post-traumatic stress disorder (PTSD), and developmental difficulties have been observed (Eth & Pynoos, 1994; Hardesty, Campbell, McFarlane, & Lewandowski, 2008; Harris-Hendriks, Black, & Kaplan, 2000), in line with reactions to other major traumatic loss reported in the broader literature (e.g. Boelen & Smid, 2017; Christ, Siegel, & Christ, 2002; Lenferink, de Keijser, Smid, Djelantik, & Boelen, 2017; Miller, 2009). While clinical experience suggests that a substantial number of children require long-term mental health and social services, little empirical evidence is available regarding the circumstances and outcomes of children bereaved by parental intimate partner homicide. A systematic search for peer-reviewed studies into psychosocial outcomes of children bereaved by domestic homicide resulted in 13 studies worldwide, the majority American case studies or case series published before 2000 (Alisic, Krishna, Groot, & Frederick, 2015). Although these studies demonstrate children’s mental health difficulties and a range of negative outcomes across social, psychological, academic, and physical domains, they also showed a striking absence of children’s own voices on their daily life, circumstances, and needs post-homicide.

The UN Convention on the Rights of the Child (United Nations, 1989) stipulates that children should have the opportunity to express their views on matters that affect them. After parental intimate partner homicide, ‘new’ adults (professionals, new caregivers) step in, shaping children’s lives with regard to living arrangements and guardianship, communication about the homicide, mental health care, and contact with the perpetrating parent in prison. A growing body of evidence shows that these types of decisions and context factors surrounding a traumatic loss have a substantial influence on eventual mental health and well-being outcomes (e.g. Brent, Melhem, Masten, Porta, & Payne, 2012; Christ et al., 2002). However, the required participation of children in decision making in the context of domestic violence is lagging behind. Callaghan, Fellin, Mavrou, Alexander, and Sixsmith (2017, p. 3371) state that ‘the failure to talk to children and young people about their lived experiences of domestic violence underestimates their capacity for agency,’ running the risk of overlooking opportunities to provide emotional and other support. Children’s own perspectives need to be part of the evidence base to inform the complex decisions following parental intimate partner homicide.

In a rare qualitative study on bereavement due to domestic homicide during adolescence, Steeves, Parker, Laughon, Knopp, and Thompson (2011) asked American adults aged 29–60 years to reflect on their life. Many participants reported a history of child abuse (before and after the homicide), as well as vivid memories of the homicide, when reflecting on their childhood. Most participants reported difficulties with intimate relationships, legal problems, and substance use in their adult lives. The youngest participant looked back at least 10 years and the oldest at least 41 years. As perspectives may change over time and retrospective accounts are known to be coloured by participants’ more recent experiences (e.g. McNally, 2003), it is also of importance to study the role of the homicide in children’s lives while they are still young.

The aim of the current study was to better understand children’s and young people’s views on their life after parental intimate partner homicide. In particular, we were interested in how they experienced their living arrangements, social environment, and general well-being.

## 2. Methods

This qualitative interview study was part of a larger project regarding the characteristics, circumstances, and well-being of children who had been bereaved by parental intimate partner homicide in the Netherlands between 1993 and 2012 (Alisic et al., 2017, 2015), approved by the University Medical Center Utrecht Ethics Committee (13/609, 24-12-2013).

### 2.1. Participants

Eligible for this study were children, adolescents, and young adults aged 8–25 years who had lost a parent due to intimate partner homicide when they were younger than 18 years old. We recruited potential participants via three routes. First, we invited eligible clients of the Psychotrauma Centre of the Wilhelmina Children’s Hospital, a nationwide tertiary mental health-care provider. Secondly, we extended invitations to potential participants via our collaborating partners (e.g. the Child Care and Protection Board, youth services). Thirdly, we asked victim support associations and associations of professionals to distribute information about the study (e.g. via their newsletter) and invite people to contact us. For all participants up to 18 years of age, we obtained written consent from the legal guardians before approaching the children and/or non-guardian caregivers for their assent. Young adults provided written consent autonomously. Participation in the study was entirely voluntary and could be ceased at any time.

Because of the multipronged recruitment approach, it was not possible to assess participation rates for the full group of participants. However, as an indication, we considered the subgroup of 60 eligible children bereaved between 2003 and 2012, whom we actively invited. We managed to reach the guardians of 51 children. For seven children, the guardians felt that they were doing reasonably well but were concerned about a potential relapse; 10 children were not coping well and therefore did not participate; for 10 children the reason for non-participation was unclear; for eight children only adults participated in the study as informants but not the children themselves; and 16 children participated themselves (27% of the children invited and 31% of those for whom a guardian was reached). Participants received a 10 euro voucher after their participation as a thank you (this was not mentioned during the informed consent procedure).

In total, 23 young people (15 female, eight male) from 14 families participated in the qualitative interviews. Their ages ranged from 8 to 24 years old, with a mean of 14 years (SD = 4; 17 children and six young adults). The homicide had happened between 18 months and 18 years earlier (mean = 8 years, SD = 5 years). All children had been born in the Netherlands (for four of them, one parent had been born abroad), and all but two had lost their mother in the homicide. For 17 children, we had information about where they were at the time of the homicide: seven children had witnessed the homicide or the crime scene, seven children were at the same place but their exact exposure was unclear, and three children were certainly not on location.

Three participants met criteria for PTSD at the time of the interview, while 12 reported some PTSD symptoms and eight did not experience PTSD symptoms [measured with the Dutch versions of the Anxiety Disorders Interview Schedule for DSM-IV (Siebelink & Treffers, 2001) or the Structured Clinical Interview for DSM Disorders (Groenestijn, Akkerhuis, Kupka, Schneider, & Nolen, 1999), depending on participants’ age; see Alisic et al., 2015, for details]. One of the children with PTSD also had clinically significant grief symptoms [measured with the Dutch version of the Inventory of Traumatic Grief (Boelen, de Keijser & van den Bout, 2001) and the Inventory of Prolonged Grief for Children and Adolescents (Spuij et al., 2012)].

### 2.2. Interviews

Three qualified mental health professionals (master’s degree level; HS, TS, AG) conducted the interviews at participants’ homes. The interviews were semi-structured, guided by a ‘topic list’ (available from the authors). The questions were based on a review of the literature (including Hardesty et al., 2008; Harris-Hendriks et al., 2000; Steeves & Parker, 2007; Steeves et al., 2011; Van Nijnatten, 2004) and tailored to each young person’s developmental level. The items covered experiences related to psychosocial development, placement, contact with the perpetrating parent, custody/guardianship, the role of relatives, the role of professional organizations, helping factors, and contact with people who had also lost a family member due to homicide, as well as expectations of the future. We reached partial saturation; while the bigger topics saturated, we still acquired new information on subtopics during our final interviews. With one exception due to a crash of the recording device, all interviews were audiotaped. The length of the interviews ranged from 18 to 68 min, with a mean of 35 min (SD = 13 min).

### 2.3. Analyses

All interviews were transcribed verbatim (with notes recorded for the interview for which the audio was lost). Our analysis was predominantly thematic. We summarized each interview with regard to the following aspects: placement, contact with perpetrating parent, guardianship, role of services, role of family, helping factors, identity and future, and psychological well-being (AG, HS, TS, using Excel spreadsheets). We subsequently made a synthesis of the summaries per topic, by exploring similarities and differences across the individual summaries – where necessary referring back to the original interviews – and selected relevant quotes to illustrate key findings (EA, AG, HS, TS). During this analysis, findings emerged within three broad categories: children’s perspectives on themselves, on their biological parents (with a focus on the perpetrating parent), and on their broader (family) environment. We discussed our final descriptions of these categories in the full team until we reached consensus. Although somewhat unusual for qualitative analysis, where it was possible to give an indication of the frequency of certain viewpoints, we did so in order to establish the extent to which a theme was a shared experience for the participants.

## 3. Results

There was substantial variation in children’s perspectives; we found both positive and negative stories on almost any topic. In that sense, an over-arching theme of ‘unique perspectives’ emerged. We describe children’s viewpoints on the three main spheres they talked about: themselves, their biological parents, and their wider (family) environment.

### 3.1. Children’s perspectives on themselves

When asked to describe themselves or when spontaneously doing so, children rarely involved the homicide explicitly. Most described themselves in a positive way, using words such as nice, strong, and friendly, and referred to themselves as someone who makes the most of things, has stable friendships, is down-to-earth, or is ‘normal, just like other kids.’ Some children also included less positive descriptors, such as being easily irritated, naughty, and impulsive, but these did not seem to have strong emotional connotations. Overall, the mostly positive self-views came with age-appropriate thoughts about the future. For example, a young boy wanted to become a millionaire, while older children described their plans regarding school, studies, jobs, travel, and family life. Nevertheless, several children also expressed some concern in their identity descriptions, such as being a ‘worrier,’ and a few had a very negative self-image. They described themselves as wearing a mask, feeling stupid, being full of uncertainty, or as ‘that girl whose dad killed her mom.’

In 18 interviews, children’s current well-being was explicitly discussed. Seven children felt they were doing well, while four children struggled significantly (with one child contemplating suicide), and seven children reported varying degrees of difficulty in between those two extremes. The struggles that the young people described included various fears and classical post-traumatic stress symptoms such as having intrusive thoughts about the homicide and avoiding reminders. They also talked about feelings of guilt over not having been able to save their parent, depressive symptoms, a negative self-image (as described above), and having difficulty trusting others.

It’s now been over two years, and I feel that I actually struggle a lot more with it than I actually thought. (…) You think ‘Oh, I can cope with it and it’s okay, because I have enough friends’ but eventually it’s on your mind a lot and you suffer from it.

Some children described that they were doing better than before. For example, one girl explained that, previously, she did not dare to talk about her sad and angry feelings, but that therapy had helped her do that. Another participant described that she had gone through some tough times but was now finally feeling better. A third example was a participant whose antidepressant medication dosage was being reduced. One participant pointed out that a trip abroad had been helpful. Her quote below brings identity and well-being together, including the wish to be ‘normal,’ which was expressed by several participants:

That helped a lot, that nobody knows you [there]. Nobody asks about it or that you have to talk about it. At home, everybody asked ‘Are you sure you’re okay?’

### 3.2. Children’s perspectives on their biological parents

The topic list for our interviews focused more on the perpetrating parent than on the victim parent. Nevertheless, many children talked about their deceased parent and had kept photographs, jewellery, or other belongings from their parent. Some children explicitly said that having those belongings was helpful or regretted not having more. One child had missed his deceased parent badly at a recent milestone when he received a school diploma. Another child looked at the picture of her mother to find out how much they looked alike. She appeared to wish for some resemblance, as she talked about similarities in their hair and teeth. A few children said that belongings did not matter so much to them; a young boy explained that the reason was that he was so young when his mother was killed. Another young person made a similar comment:

Sometimes it feels unreal. ‘Has it really happened?’ I ask myself then, ‘I don’t know either of my parents, I have never seen them.’

Regarding the perpetrating parent, children also expressed varying views, although these were mostly negative. Several children described that they never wanted to see their parent again. One child described his father as his worst enemy, another said he could ‘go to hell,’ and a third referred to his father only as ‘the fool.’ Other children were less strong in their expressions, and one girl expressed a struggle reconciling two sides of her father:

In the past, my father was actually a very good person for me because he always went horse riding with me and such things. And then suddenly the man I most, my father simply, who I trusted most and who I loved most, had done something very nasty.

Siblings did not necessarily have the same viewpoints of their perpetrating parent or make the same decisions. For example, some children opted to visit their parent in prison, while their siblings had decided to cease contact.

In some cases, participants’ stories conveyed that perpetrating parents were threatening in their words or behaviour. They mentioned fear that their parent might kill them, their caregivers, or their friends. Several parents who had been released or were on leave had shown up without warning. Even when they were not violent, this caused distress and feelings of unsafety. One child described that their parent had requested photographs, which they did not want to be available to him (a judge eventually decided not to allow the perpetrator to receive the pictures). While most perpetrators were fathers, one of the children whose mother had killed was also afraid of her. The child described that she was currently ‘learning not to kill anyone.’

The children had various types and amounts of contact with the perpetrating parent; from no contact to infrequent postcards or phone calls to regular prison visits. The young people mentioned various reasons for having contact. These included the opportunity or wish to ask questions (e.g. about the victim parent), checking resemblance with the parent (e.g. the desire to have a one-off meeting, hoping that there would not be any resemblance), joining a sibling, hearing remorse, or answering a wish from the perpetrator to have contact (e.g. one child wondered out loud whether she had had contact just for the sake of the parent and whether she should be ending it). Having contact for the purpose of keeping or building a connection was rarely mentioned.

Most children said that they had the freedom to decide whether or not they wanted to have any contact with the perpetrator parent, and were content with the current arrangement. Several mentioned that they felt supported by their caregivers and professional guardians in their decisions. In a few cases, the children felt that their caregivers or professional guardians had made the decision rather than themselves, or preferred them to make the decision:

When I have a good day, I like to do it myself, and when I have a bad day, I don’t like to decide.

Some children described that they had had contact initially but that it had affected them negatively (e.g. experiencing sleeping problems after visits) and that they had therefore ceased contact. Others were contemplating contact in the future. In several cases, children described that they preferred to have someone (e.g. guardian or caregiver) with them when they met their parent. One girl described that she preferred not to sit next to her parent. Another child explained that she preferred her father to stay in prison:

I would have liked it better if he stayed in prison and that I could go there and then leave again. Because you go there and … he sits behind kind of, he sits in the same room … behind bars. Then he is just there, and I feel safer.

### 3.3. Children’s perspectives on their broader (family) environment

In all interviews, children’s living arrangements were discussed. At the time of the interview, nine children lived with family of the victim, two with family of the perpetrator, four with acquaintances of both parents, one with acquaintances of the victim, three in a neutral foster placement, one in a residential care setting, and three independently. Ten children talked about some concerns in their (previous) placements. Most children had moved once or several times, sometimes also between family ‘sides.’ The reasons for the moves included caregiver capacity, developments in legal procedures (e.g. being placed back with the father after his acquittal, only to be re-placed after a subsequent conviction), behavioural difficulties, or difficulties in connecting with new family members. Again, siblings did not necessarily experience these situations similarly, and sometimes these issues led to their separation:

I was sad that my little brother had to leave, I would have liked to go with him but that wasn’t possible. I had settled a bit already here and I thought ‘I don’t want to move again so I’ll stay here.’

While several children had been separated from their siblings and would have preferred to stay together, one child felt that he would probably still choose his current situation (staying at the same school) over staying together. Children mentioned their siblings regularly as a source of strength and support.

Thirteen participants were generally happy with their placement. Some children remarked that they were glad they were placed with people they knew and trusted. One child felt that her new neutral foster family had probably given her opportunities that she otherwise would not have had. Several young people felt that they had had a good childhood at their placement.

The relationship with family members other than the caregivers, and between the two sides of the family, was discussed in virtually all interviews (*n *= 22). Ten participants described these relationships as positive or satisfactory. They spoke about either having good contact with both sides of the family, or no longer having contact and being fine with it.

Actually, the whole family of my father has made a decision, like ‘We are here for the children, they need to be happy.’ That makes you feel very much supported.

Several children among those with satisfactory relationships emphasized that family was very important for them, although one young person also explained that he preferred no longer having contact, in order to be able to move on. Ten other participants had mixed experiences, including some conflict or lack of safety, and two had very negative experiences, involving strong aggression. In a few cases, the children saw their family members under guidance.

Other actors that came up in children’s descriptions were friends, teachers (both seen as helpful and sometime as asking too much), and the mental health and social services professionals involved. Most young people were positive or neutral with regard to these services. They were pleased to be asked for their opinion regarding decisions, and one participant mentioned that she was very happy with the support she received regarding contact arrangements. Therapeutic approaches, including eye movement desensitization and reprocessing, play therapy, neurolinguistic programming, trauma therapy, and conversations with placement workers, were described as sometimes difficult but helpful. Participants mentioned that they found out that they were not alone and that they could learn to work through emotional problems.

[Therapy] was a challenge, it was. You are of course talking about yourself each time and sometimes I find it difficult to talk about myself. But eventually, I’ve gained a lot from it and I’m happy with that, that I did it.

Seven participants had criticisms or negative experiences with regard to professionals. One criticism was that too many different organizations were involved and that it was overwhelming (although there was also a child who stated the opposite, that the youth services/guardian did not show up often enough). Participants’ stories also showed a lack of continuity, with multiple changes in guardians, youth services, or placement workers. One young person estimated that she had had six different guardians. One child felt that her (family’s) agency was restricted by the child protection board and youth services; they made too many decisions for them. One child said that they did not see the benefits of talking with mental health professionals.

In the 12 interviews in which contact with peers who had experienced domestic homicide was discussed, only one participant was having such contact. Five said that they would not be interested, two said that they would be interested, three did not specify whether or not they would want such contact, and one participant did not feel strongly about it either way. The child who interacted with other young people bereaved by domestic homicide felt that it was helpful and supportive.

## 4. Discussion

The young people in our study conveyed a range of experiences and opinions regarding their circumstances after parental intimate partner homicide. While most were fairly content with themselves and their living arrangements, they expressed also several substantial and persistent difficulties, including distress, conflicts between family members, and feelings of unsafety. Overall, children’s viewpoints varied considerably, even between siblings. This unique nature of children’s perspectives is an important consideration for professionals involved after a homicide. On the one hand, it underlines children’s capacity to make up their own mind about key aspects of their life. On the other hand, it reinforces the need to explore these viewpoints since it is unlikely that our expectations about a child’s needs or perspectives are accurate without asking them directly.

Before further interpreting the results, it is important to realize their limitations. Most importantly, the young people participating in this study are unlikely to be representative of all children bereaved by domestic homicide. Guardians who thought that potential participants were not coping or were at risk for a relapse usually did not proceed to invite the children for participation; the children in our sample were probably doing better than average. Also, for most participants the homicide had been several years ago. They would probably have shown different distress profiles had we conducted the interviews within, for example, 2 years post-homicide. In addition, the percentage of immigrant children [born abroad or their parent(s) born abroad] was substantially lower than in the population of children exposed to domestic homicide (17% vs 59%; Alisic et al., 2017). It is unlikely that we can understand the experiences of children from immigrant families through the current data. More generally, our findings are unlikely to generalize to other countries and cultures, since different social, economic, cultural, and child-protection contexts may shape children’s trajectories and perspectives differently (in the future, combining data from various countries may also allow for more in-depth analyses, e.g. regarding the country differences mentioned, but also regarding developmental and other differences). Finally, our interviews were relatively short to limit the burden on the children, and therefore, we focused on their current circumstances and, for example, did not ask them in depth about their deceased parent or discuss displays of resilience over time. We have caught only a fraction of the stories that they might have told us if we had had the time for multiple interactions. Despite these limitations, the current findings contribute to the inclusion of children’s voices regarding highly sensitive and complex aspects of their life after parental homicide.

The fact that children’s viewpoints on many topics differed is in line with the variability in grief reactions found more broadly in children who have been bereaved (Christ et al., 2002; Miller, 2009). In the context of parental intimate partner homicide, this underlines that children are capable of developing their own opinions and expressing their wishes. We see this as both a strength and something that professionals should seek to facilitate and reinforce where possible. The homicide and all its consequences, including having to move house, are the opposite of ‘being in control.’ Supporting children to have their say in, for example, living and contact arrangements seems, therefore, to be crucial, taking into account that siblings may have different perspectives. Of course, facilitating children’s own voice and influence does not happen in a vacuum, and as the children recounted, there are sometimes (violent) conflicts between members of their family which may limit the possibilities or pressure children to be loyal in a way that may conflict with their own views. This is a complex situation to navigate, and, unfortunately, no other solution exists than careful listening to the children and caregivers, and attempting to mediate any conflicts before or when they arise.

Despite the substantial variation in young people’s viewpoints, a few trends within the interviews stood out for us. First, several children referred directly or indirectly to the wish to be (seen as) normal. This wish to be just like other children has been observed in other qualitative child trauma research as well (e.g. Alisic, Boeije, Jongmans, & Kleber, 2011; Urman, Funk, & Elliott, 2001). Apart from questions regarding alienation, this desire to be just like other kids is usually not part of the quantitative measures related to child traumatic stress or traumatic grief. It may be valuable to explore this theme in more depth and to investigate how children may get the opportunity to feel normal after a parental intimate partner homicide, without ignoring their reality.

Secondly, it struck us that a wish to build and maintain a relationship with the perpetrator parent was often absent from these young people’s narratives. Reasons for remaining in contact with the parent were predominantly other than a desire for maintaining the relationship as such, and some children appeared to just go along with the arrangement in place, although a few mentioned that they were content with it. Clinically, however, we have also encountered children who expressed a wish to be in contact because they missed their parent. This requires further exploration. Among adults around the children, we sometimes hear blank statements regarding what would be best for children regarding contact with the perpetrator. Whatever children’s reasons are for their stance, in our view they should never be forced into having contact if they do not want it, or denied contact if they wish for it and it is feasible.

Thirdly, even though for many children the homicide had been several years ago, there were still substantial concerns regarding safety. These occurred in the context of contact with the perpetrating parent as well as conflict between family members. In this case, one cannot safely assume that the risks are low, because there has evidently been a fatality before. Areas to explore further include how to ensure that children feel safe in their contact with the perpetrating parent (if they want that contact) and whether mediation between families as a standard offer in these specific cases may be of use.

Finally, while children bereaved by domestic homicide are confronted with extensive instability over long periods, stability and continuity appeared to be important supportive factors. Although we would not want to advocate for yet another professional to be added to the list of care providers to involve, it seems important that children have one stable, trusted person to go to with any questions and concerns (Steeves, Laughon, Parker, & Weierbach, 2007). Professionals involved could enquire who this person might be. For example, they could be a guardian, a teacher, a counsellor or therapist, or possibly a family member if they are not affected by the homicide themselves; it would need to be someone who will be available to the child for multiple years.
